# Quantitative Comparison of a Handheld and a Table-Top Fundus Camera for Retinal Microvascular Assessment

**DOI:** 10.3390/reports9020147

**Published:** 2026-05-11

**Authors:** Lazaros K. Yofoglu, Georgios Zervas, Christina Konstantaki, Chrysoula Moustou, Evaggelia K. Aissopou, Petros P. Sfikakis, Irini Chatziralli, Kimon Stamatelopoulos, Athanase D. Protogerou, Antonios A. Argyris

**Affiliations:** 1 Cardiovascular Prevention and Research Unit, Clinic/Laboratory of Pathophysiology, School of Medicine, National and Kapodistrian University of Athens, 115 27 Athens, Greece; 2 Angiology and Endothelial Pathophysiology Unit, Department of Clinical Therapeutics, School of Medicine, Alexandra Hospital, National and Kapodistrian University of Athens, 115 28 Athens, Greece; 3 1st Department of Propaedeutic and Internal Medicine, School of Medicine, Laiko Hospital, National and Kapodistrian University of Athens, 115 27 Athens, Greece; 4 2nd Department of Ophthalmology, School of Medicine, Attikon Hospital, National and Kapodistrian University of Athens, 124 62 Athens, Greece; 5 Hellenic Foundation for Cardiovascular Health and Nutrition, 155 27 Athens, Greece

**Keywords:** CRAE, CRVE, AVR, Optomed Aurora, Topcon TRC-NW8, fundus camera

## Abstract

**Objectives:** The aim of this study was to compare a widely applied table-top digital non-mydriatic camera (Topcon TRC-NW-8) with a handheld digital non-mydriatic camera (Optomed Aurora IQ) regarding the quantitative assessment of the retinal microcirculation using established biomarkers: central retinal arteriolar equivalent (CRAE), central retinal venular equivalent (CRVE) and arterio-venous ratio (AVR). **Methods:** The present cross-sectional study included 26 randomly selected participants (51 eyes) who underwent retinal imaging of both eyes with the two devices and were analyzed using a static retinal vessel analyzer. **Results:** The mean differences in CRAE, CRVE and AVR between the two devices (Topcon/Aurora) were 24.96 ± 11.7, 22.7 ± 11.7 and 0.026 ± 0.045, respectively. Strong correlations were observed between devices (r = 0.84 for CRAE, 0.75 for CRVE and 0.83 for AVR; all *p* < 0.001), with high agreement as indicated by ICC values (0.91, 0.85, and 0.90, respectively). Bland–Altman plots indicated evidence of systemic bias (95% within 2 SD) with no proportional bias, as the differences were consistently distributed across the range of average values. Regression-based equations were derived to approximate the transformation of measurements between devices. **Conclusions:** The handheld fundus camera demonstrates strong correlation and good relative agreement with the table-top device; however, a consistent device-dependent bias limits the direct interchangeability of absolute measurements. The derived transformation equations may facilitate approximate cross-device comparison, although external validation is required. These findings support the complementary use of handheld devices and highlight the need for calibration strategies when integrating measurements across platforms.

## Highlights

- Lack of comparative quantitative studies between handheld and table-top fundus cameras.
- Strong correlation and good agreement in terms of CRAE, CRVE and AVR assessment between the two cameras.
- Handheld camera shows systematic underestimation with consistent bias.
- Devices may be used complementarily or possibly interchangeably after calibration in research and clinical fields.

## 1. Introduction

Retinal microcirculation is an important and intricate vascular network of the human body. Its assessment is highly valuable for both clinical and research purposes, as it not only allows quick, easy and non-invasive in vivo observation of the human central nervous system microcirculation [1] but is also indicative of findings in other microvascular beds, such as those of the heart [2,3], brain [4] and kidneys [5], which share similar anatomical and functional characteristics.

Ocular fundus photography enables direct visualization and qualitative as well as quantitative assessment of retinal microcirculation. Qualitative assessment is considered an important tool for evaluating clinical manifestations and thus for the diagnosis and treatment strategies of several ocular diseases, such as hypertensive retinopathy, diabetic retinopathy, vascular occlusion, etc. This quantitative assessment involves the computer-based measurement of retinal vessel diameters greater than 35–40 μm [6] and the calculation of the central retinal arteriolar equivalent (CRAE), central retinal venular equivalent (CRVE) and arterio-venous ratio (AVR) using the revised formulas of Hubbard and Parr [7]. The literature suggests a possible superiority of quantitative assessment over the qualitative assessment in detecting the early onset of retinal microcirculation changes [8]. Particularly, retinal vessel diameter alterations and the presence of arterial stenosis yield a greater prognostic value for cardiovascular disease (CVD) risk assessment, while clinically overt manifestations, such as retina hemorrhages, cotton wool spots or optic disc swelling, have later onset, limiting their prognostic significance in primary prevention [8].

A widely applied methodology for the quantitative assessment of retinal microcirculation includes the use of digital table-top non-mydriatic cameras. However, their size, weight and cost limit their large-scale use [9]. Portable non-mydriatic handheld retinal cameras may represent a possible cost-effective alternative and because of their small size can enable the provision of eyecare services in remote areas where poor referral and difficulties in transportation may result in a breakdown of care, increased costs, or delayed treatment [10]. These devices are already broadly used for the qualitative assessment of the retina with similar accuracy to table-top cameras [11]. Therefore, the aim of the present cross-over study was to compare a widely applied table-top digital non-mydriatic camera (TOPCON NW-8) with a handheld digital non-mydriatic camera (Optomed AURORA) in terms of quantitative assessment of the retinal microcirculation via the most commonly used vessel diameter biomarkers.

## 2. Materials and Methods

The present cross-sectional study was based on a predefined protocol registered to the Cardiovascular Prevention and Research Unit, Clinic/Laboratory of Pathophysiology, School of Medicine, National and Kapodistrian University of Athens, Athens, Greece.

All the definitions and methodology that were applied in order to assess the retinal vessel calibers using the CRAE, CRVE and AVR are described in the Supplementary Materials. The data that were analyzed in this study are available from the corresponding author upon reasonable request.

### 2.1. Study Design–Population

The present cross-sectional study included twenty-six consecutively recruited participants from the outpatient clinic and the research staff of the Cardiovascular Prevention and Research Unit/Clinic and Laboratory of Pathophysiology, National and Kapodistrian University of Athens. Prior to the present study, a pilot study was conducted involving 5 participants (10 eyes). Based on a power analysis—assuming a power of 0.80, an alpha level of 0.05 (two-tailed), and a projected difference in retinal vascular diameter values of approximately ~24 μm between the two devices—it was determined that a minimum of 33 eyes was required. This sample size is similar to the mean sample sizes in the literature. The protocol of the present study has been approved by the Bioethics Committee of the Medical School of the National and Kapodistrian University of Athens. Participants provided written informed consent according to the World Health Organization statement on ethical principles for medical research involving human subjects, developed in Helsinki [12], and the protocol was approved by the “Laiko” Hospital’s institutional review board.

### 2.2. Inclusion/Exclusion Criteria

The present cross-sectional study included adults (above 18 years old) without cataracts, eye affecting genetic disorders, and retinal disorders, such as ablatio retinae or macular degeneration, and those free from any eye drop medication.

### 2.3. Analysis of the Retinal Microcirculation

All participants were asked to refrain from eating food, any vasoactive substance (smoking or alcohol), medication and physical activity at least 3 h before examination. Before the examination, participants’ medical history was obtained with special emphasis on ophthalmologic history. Acquisition of all images was performed by the same experienced examiner. Both eyes of each participant were photographed with a 45° digital non-mydriatic static retinal camera (Topcon TRC-NW8, Tokyo, Japan) and a digital non-mydriatic portable retinal camera (Optomed Aurora IQ, Oulou, Finland) after 5 min of adaptation in a dark room (25 degrees Celsius). For each participant, the camera order was randomly selected to avoid systematic bias. Two fundus photographs were obtained for each eye per device: a disc-centered image and an image in the primary (natural) position, with a 3 min adaptation in the dark after each shot. To ensure data integrity, no duplicate images of the same view were acquired. From the total of four photographs obtained for each participant, only the disc-centered ones for each eye were quantitatively assessed and analyzed by a well-trained physician blinded to clinical data (L.Y.). For each photograph, the calibers of retinal arterioles and venules passing through a zone between 0.5- and 1.0-disc diameters from the optic disc margin were measured and analyzed using a Static Retinal Vessel Analyzer (SVA-T and Vesselmap 2 software, Visualis, Imedos Systems UG, Jena, Germany) [13]. These measurements were then summarized using formulas given by Hubbard et al. [14] and if required, modified by Knudtson et al. (“Big 6 tool”-only 6 vessels are used for the measurements) [7] to compute CRAE and CRVE, representing the average internal caliber of retinal arterioles and venules, respectively. Afterwards, CRAE and CRVE were used to estimate AVR. Lastly, the retinal images with the eye in a natural position were qualitatively assessed by a well-trained physician blinded to clinical data (L.Y.).

### 2.4. Statistical Analysis

Statistical analysis was performed using the SPSS statistical package (IBM, version 29.0; IBM, Armonk, NY, USA). Normality of the continuous variables was tested using the Kolmogorov–Smirnov test and a visual inspection of the Q-Q plots. Normally distributed continuous variables are presented as mean values ± standard deviation (SD) and categorical variables as numbers and percentages. Multiple linear regression analysis was performed to determine the relationship of age, gender, body mass index (BMI), smoking, coffee consumption, alcohol intake, exercise, hypertension (HTN), diabetes mellitus (DM) and retinal camera used (Topcon NW-8 or Optomed Aurora) with retinal vessel caliber biomarkers (CRAE, CRVE and AVR), adjusted for fellow caliber to provide unbiased and biologically plausible results as suggested by Liew et al. [15]. Additionally, regression models were used to derive equations enabling the approximate conversion of retinal vessel measurements between devices. Results are presented as standardized and unstandardized beta coefficients and 95% confidence intervals (CIs). Each eye was evaluated as an independent unit of observation, as ocular pathology—such as hypertensive retinopathy—can manifest asymmetrically despite systemic conditions, so for the purposes of the present statistical analysis, each eye of the participants was considered a separate entity and adjustment for the opposite eye was performed. Consequently, our regression models treated each eye as a distinct entity while adjusting for inter-eye correlation within the same patient.

The comparison between measured retinal indices of the two devices was performed using (i) their mean difference, (ii) correlation coefficients, (iii) intraclass correlation coefficient (ICC) (ICC model was based on consistency; it was two-way, and the average measures were analyzed) and (iv) Bland–Altman plots. All tests were two-sided, and the level of statistical significance was set at *p* < 0.05 [16].

## 3. Results

The study population consisted of 26 adults (mean age 39.68 ± 18.12 years, 50% males). Due to one participant’s refusal, imaging of the right eye could not be performed, with the total number of eyes being limited to 51; descriptive characteristics of the study population are presented in Table 1.

The mean values of CRAE, CRVE and AVR measured with each device are presented in Table 2. The mean differences in CRAE, CRVE and AVR between the two devices were 24.96 ± 11.7, 22.7 ± 11.7 and 0.026 ± 0.045, respectively, with the static camera having greater values in all three biomarkers. These differences were statistically significant and dependent on confounders. Positive associations for each biomarker between the two cameras were found (CRAE, CRVE and AVR: 0. 843, *p* < 0.001; 0.754, *p* < 0.001; 0.826, *p* < 0.001, respectively). A high level of correlation for all three biomarkers was noted, as indicated by the ICC (0.913 for CRAE, 0.853 for CRVE and 0.902 for AVR).

In the regression analysis (Table 3), CRAE had a statistically significant positive association with diabetes (β = 0.264; *p* = 0.026) but also a negative association with BMI (β = −0.260; *p* = 0.021), alcohol consumption (β = −0.235; *p* = 0.007), HTN (β = −0.311; *p* = 0.011) and the type of camera used (−0.523; *p* < 0.001) (fully adjusted model for confounders). A statistically significant positive association was observed for CRVE with gender (β = 0.194; *p* = 0.042), exercise (β = 0.181; *p* = 0.026), and diabetes (β = 0.363; *p* = 0.002) and a negative one with HTN (β = −0.259; *p* = 0.032) and the type of camera used (−0.575; *p* < 0.001) (fully adjusted model for confounders). AVR had a statistically significant negative association with BMI (β = −0.269; *p* = 0.033), alcohol consumption (β = −0.488; *p* < 0.001), exercise (β = −0.245; *p* = 0.008) and the type of camera used (−0.177; *p* = 0.036) but also a negative trend with HTN (β =−0.241; *p* = 0.078) (fully adjusted model for confounders). A more detailed presentation of regression analysis for each biomarker can be found in Tables S1–S3 in the Supplementary Materials.

All the above results can be summed up in three equations for each retinal vessel diameter biomarker:

CRAEtopcon = 28.907 − 0.099 × Age + 10.364 × Gender − 0.049 × BMI − 2.529 × Smoking + 0.208 × Caffeine − 8.479 × Alcohol − 5.480 × Exercise + 10.268 × HTN + 4.520 × DM + 3.561 × Eye + 0.919 × CRAEaurora R square: 0.816 (Statistically significant for: BMI, Alcohol, CRAEaurora)

CRVEtopcon = 60.975 + 0.019 × Age + 5.885 × Gender + 0.383 × BMI + 6.404 × Smoking − 4.903 × Caffeine − 0.917 × Alcohol − 2.472 × Exercise + 5.985 × HTN − 3.518 × DM + 1.329 × Eye + 0.710 × CRVEaurora R square: 0.686 (Statistically significant for: CRVEaurora)

AVRtopcon = 0.146 − 0.001 × Age + 0.023 × Gender − 0.002 × BMI − 0.034 × Smoking + 0.022 × Caffeine − 0.026 × Alcohol − 0.005 × Exercise + 0.010 × HTN + 0.062 × DM + 0.10 × Eye + 0.895 × AVRaurora R square: 0.767 (Statistically significant for: Smoking, AVRaurora)

The variables Age, BMI, CRAEaurora, CRVEaurora, and AVRaurora are quantitative, while the rest are binary. These equations were derived from a sub-analysis of all 51 eyes that were included in the study.

**Figure 1:**
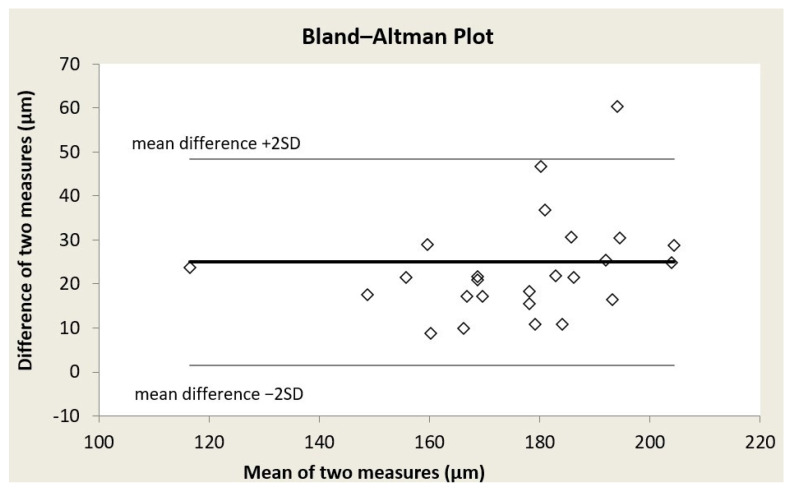
Bland–Altman plot for central retinal arteriolar equivalent (CRAE).

**Figure 2:**
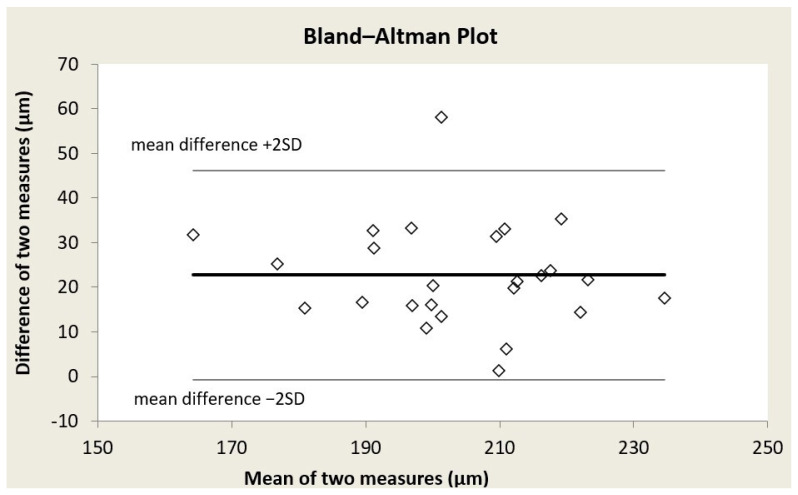
Bland–Altman plot for central retinal venular equivalent (CRVE).

**Figure 3:**
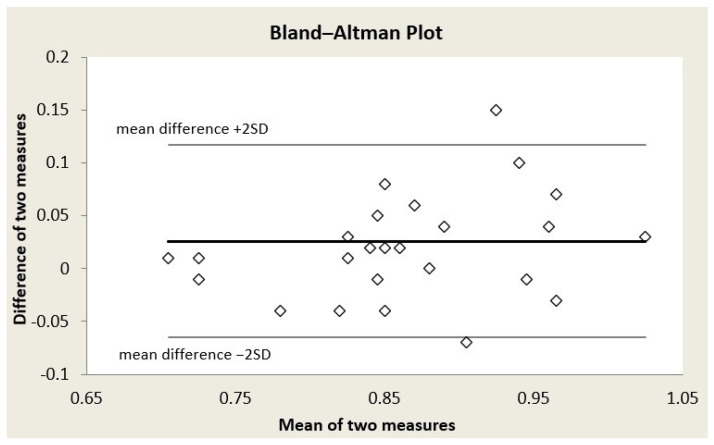
Bland–Altman plot for arteriolar-to-venular diameter ratio (AVR).

Bland–Altman plots were formed for each of the three retinal vessel diameter biomarkers (Figure 1, Figure 2 and Figure 3); acceptable limits of agreement across all three indices are shown. Bland–Altman analysis revealed for CRAE, a mean bias of 24.96 ± 11.72 μm (95% limits of agreement from 1.53 to 48.4 μm); for CRVE, a mean bias of 22.70 ± 11.68 μm (95% limits of agreement from −0.66 to 46.07 μm); and for AVR, a mean bias of 0.03 ± 0.05 (95% limits of agreement from −0.07 to 0.12) (in all three cases, the mean difference is Topcon minus Aurora). While a systematic bias was observed, the differences were consistently distributed across the range of average values, indicating no proportional bias.

## 4. Discussion

This is the first study to compare a novel handheld non-mydriatic retinal camera with a classic table-top retinal camera, regarding the quantitative assessment of retinal microvascular biomarkers in a CVD-free population. The main finding is that the CRAE, CRVE and AVR measurements provided by the handheld camera showed strong correlations and high intraclass correlation coefficients with the table-top camera. However, the handheld camera consistently underestimated these indices compared with the table-top system, indicating systematic bias between devices, thereby limiting direct interchangeability for absolute vessel caliber measurements.

Previous studies focusing on qualitative outcomes have shown that the handheld camera Optomed Aurora had similar results to a table-top fundus camera in detecting and grading diabetic retinopathy, hypertensive retinopathy and diabetic maculopathy [17]. Specifically, this handheld camera had specificity above 90% and sensitivity nearly 100% in detecting diabetic retinopathy [11,17], while both specificity and sensitivity were 100% as far as hypertensive retinopathy is concerned [17]. The majority of the conducted studies summed up that this portable camera can be used for quality assessment of the retina and especially for screening diabetic retinopathy [11,17,18,19,20]. However, in the current literature, there is a lack of studies comparing a table-top non-mydriatic camera with a handheld non-mydriatic camera in the quantitative assessment of retinal vessels. To our knowledge, this is the first study to directly compare handheld and table-top non-mydriatic cameras specifically for quantitative retinal microvascular biomarkers, addressing an important gap in the literature.

In the present study, the camera type remained a significant independent predictor of all three retinal biomarkers in the fully adjusted regression model, even after accounting for major clinical and demographic confounders such as age, gender, BMI, smoking, caffeine, alcohol, exercise, HTN, DM and left or right eye. For each biomarker, the extent to which each of these confounders influences the deviation is demonstrated in the formulas presented above. This finding reinforces the fact that the observed differences are not explained by between-subject variability in traditional cardiovascular risk factors but rather represent a systematic, device-dependent measurement offset. This further argues against treating the two devices as directly interchangeable for absolute value comparisons.

This underestimation likely reflects inherent technical differences between devices, including optical design, sensor characteristics, illumination, and image magnification, and greater susceptibility to subtle misalignment during image acquisition. This difference between the two cameras was statistically significant and clinically relevant, as the mean bias for both biomarkers CRAE and CRVE was approximate at the level of 35 μm, which is prominent. In all 51 cases, AVR was either above or below 0.82 in both devices, which is considered to be the cut-off point of the normal and pathological vessel diameter ratio, respectively, as values below 0.82 are associated with increased CV risk [21]. Importantly, Bland–Altman analysis showed no evidence of proportional bias, as the slope was ≈0, suggesting that the difference between devices remains relatively constant across the range of vessel diameters (systemic bias). Additionally, three equations were derived from the regression models, enabling the approximate conversion of Aurora retinal vessel diameter biomarkers to the corresponding values of the Topcon camera. This finding supports the notion that device-specific calibration or correction approaches may allow meaningful comparisons across imaging platforms, especially useful in comparative or longitudinal studies—particularly when measurements are consistently obtained using the same device. Therefore, the good relative agreement and possible complementary use of these two devices via the derived equations could enable a possible comparison and assessment of the current CV risk on handheld acquired retinal photos using the existing literature for the table-top cameras [22].

Our results underscore the important distinction between correlation and agreement; while the devices rank individuals similarly, they do not provide identical absolute measurements. Notably, the persistence of camera type as an independent predictor in multivariable analysis supports the presence of a true device-dependent measurement effect. To address this limitation, we derived regression-based equations that allow approximate transformation of retinal vessel measurements between devices. While these equations do not eliminate the observed bias, they suggest that the relationship between devices is predictable and may be partially corrected. This approach can be particularly useful in research settings where the integration of data from different imaging platforms is required. However, these findings should be interpreted with caution, as the proposed equations are based on a relatively small sample and have not been externally validated. Therefore, the two devices should not be considered directly interchangeable for absolute measurements but rather complementary, with the potential for calibration-based harmonization in future studies.

We not only found a level of absolute bias of the measured indices between the two devices, as indicated mainly with Bland–Altman analysis, but also high relative agreement and reliability consistency. From a clinical and research perspective, this suggests that handheld cameras may be reliably used for within-study comparisons, longitudinal follow-up, or large-scale epidemiological studies, particularly when the same device is used consistently over time. However, absolute values derived from different cameras should not be considered directly interchangeable without appropriate calibration, especially when applying cut-offs derived from table-top camera-based literature. The establishment of baseline values, as far as retinal vessel diameter biomarkers are concerned, is critical for the objective assessment of retinal vessel metrics. While a prior prospective observational study has utilized confocal scanning laser ophthalmoscopy (cSLO) technology to determine normative data for both retinal arteriolar and venular caliber measurements using images from 300 participants free of systemic or ocular illness, those data cannot be directly compared with the present findings owing to disparities in imaging modalities and acquisition techniques [23].

Furthermore, beyond device comparison, the regression analysis revealed significant associations between retinal biomarkers and clinical variables, many of which align with established pathophysiological insights. Specifically, there was a statistically significant association between high BMI and lower CRAE and AVR values, HTN and lower CRAE and CRVE values, alcohol consumption and lower CRAE and AVR values, and physical exercise and higher CRVE and lower AVR values, findings consistent with the existing literature [24,25,26]. It is important to highlight the fact that, due to the small number of diabetic participants, despite the statistically significant associations between DM and retinal biomarkers, these results cannot be taken into consideration. These findings highlight the potential of retinal biomarkers as indicators of systemic vascular health.

While this study provides valuable insights, certain limitations should be acknowledged. First, the relatively small sample size reflects the exploratory nature of this investigation and is comparable to that of prior methodological studies evaluating retinal vessel measurement techniques. While this limits statistical power for extensive multivariable modeling, the primary objective of the study was to assess agreement between imaging devices rather than to establish definitive causal or prognostic associations. Second, the study population itself may limit the generalizability of the above findings, as it was relatively young and characterized by a low prevalence of cardiometabolic disease and free of ophthalmic disorders such as ablatio retinae or macular degeneration or eye drop medication. These characteristics may limit the generalizability of absolute retinal vessel values to older or higher-risk populations. Finally, quantitative analyses were performed using a single, well-established retinal vessel analysis software, and results may differ when alternative analytical platforms are applied.

## 5. Conclusions

In conclusion, the handheld non-mydriatic fundus camera demonstrated strong correlation and good relative agreement with the table-top device in the assessment of retinal microvascular biomarkers. However, a consistent device-dependent underestimation of vessel calibers indicates the presence of systematic bias, thereby limiting the direct interchangeability of absolute measurements. Importantly, the derivation of regression-based transformation equations in the present study provides a potential approach for approximating measurements between devices, which may facilitate comparative analyses across imaging platforms. Nevertheless, these equations should be interpreted cautiously and require external validation before clinical or research application. Overall, handheld devices may be reliably used for within-device comparisons, longitudinal follow-up, and settings where portability is essential, while calibration strategies remain necessary for cross-device integration.

## 6. Perspectives

The present study supports the use of handheld non-mydriatic fundus cameras as a practical tool for quantitative assessment of retinal microvascular biomarkers. The good relative agreement and complementary use observed between handheld and table-top devices highlight the potential of portable retinal imaging to extend microvascular phenotyping beyond specialized ophthalmology or tertiary-care settings. This is particularly relevant for cardiovascular risk stratification, population-based studies, and telemedicine applications, where accessibility, cost, and ease of use are paramount—especially in regional or remote areas of developing nations where limited facilities and the necessity of long-distance travel often hinder access to standard ophthalmic care. As retinal vessel calibers reflect systemic microvascular health, the ability to obtain reliable quantitative measurements using handheld devices may facilitate their integration into routine cardiovascular prevention programs and large-scale epidemiological research. This objective requires the development of a normative database based on analyzing digital fundus images, similarly to a prior effort involving images captured through cSLO [23]. Future work should focus on the development of standardized acquisition protocols and cross-device calibration strategies, enabling harmonized interpretation of retinal biomarkers across imaging platforms and enhancing their translational utility in both clinical practice and research.

## Figures and Tables

**Table 1:** Demographic characteristics of the study participants (*n* = 26).

Parameters	
Age (years)	39.69 ± 18.12
Male gender, *n* (%)	13 (50)
Height (cm)	171.81 ± 11.68
Weight (kg)	74.74 ± 18.67
Body mass index (kg/m^2^)	25.16 ± 4.89
HTN, *n* (%)	6 (23.1)
DM, *n* (%)	2 (7.7)
Dyslipidemia, *n* (%)	4 (15.4)
Renal disease, *n* (%)	2 (7.7)
CVD, *n* (%)	0 (0)
Autoimmune disease, *n* (%)	4 (15.4)
Infectious disease, *n* (%)	1 (3.8)
Smoking, *n* (%)	7 (26.9)
Smoking (years)	6.46 ± 14.53
Smoking (pack/years)	5.36 ± 12.47
Caffeine consumption, *n* (%)	21 (80.8)
Alcohol consumption, *n* (%)	13 (50)
Exercise, *n* (%)	19 (73.1)
Family history of HTN, *n* (%)	13 (50)
Family history of DM, *n* (%)	10 (38.5)
Family history of CVD, *n* (%)	9 (34.6)
Ophthalmological disease, *n* (%)	18 (69.2)

Values are expressed as means ± standard deviations or numbers (percentages) as appropriate. Abbreviations: HTN, hypertension; DM, diabetes mellitus; CVD, cardiovascular disease.

**Table 2:** Comparison of CRAE, CRVE and AVR between Topcon and Aurora devices, coefficient correlation (r) and intraclass correlation (ICC) for each index between the two devices.

	Topcon	Aurora	*p*	r	*p*	ICC (95% C.I.)
**CRAE (μm)**	190.38 (21.51)	165.42 (19.93)	<0.001	0.84	<0.001	0.91 (0.85 to 0.95)
**CRVE (μm)**	215.57 (14.91)	192.87 (17.59)	<0.001	0.75	<0.001	0.85 (0.74 to 0.92)
**AVR**	0.88 (0.08)	0.86 (0.07)	<0.001	0.83	<0.001	0.90 (0.83 to 0.94)

Values are expressed as means (standard deviations). CRAE, central retinal arteriolar equivalent; CRVE, central retinal venular equivalent; AVR, arterio-venous ratio.

**Table 3:** Regression analysis with CRAE, CRVE and AVR as dependent variables (fully adjusted model).

Factors	CRAE		CRVE		AVR	
	Stand. β	*p*-Value	Stand. β	*p*-Value	Stand. β	*p*-Value
Age	−0.122	0.291	−0.121	0.289	−0.040	0.755
Gender	0.124	0.196	0.194	**0.042**	−0.026	0.808
BMI	−0.260	**0.021**	−0.118	0.283	−0.269	**0.033**
Smoking	−0.001	0.992	−0.076	0.396	−0.083	0.413
Caffeine	0.006	0.948	−0.039	0.658	0.041	0.688
Alcohol	−0.235	**0.007**	0.106	0.213	−0.488	**<0.001**
Exercise	−0.021	0.796	0.181	**0.026**	−0.245	**0.008**
HTN	−0.311	**0.011**	−0.259	**0.032**	−0.241	0.078
DM	0.264	**0.026**	0.363	**0.002**	0.035	0.790
Left or Right Eye	0.045	0.538	−0.006	0.939	0.059	0.475
Aurora–Topcon	−0.523	**<0.001**	−0.575	**<0.001**	−0.177	**0.036**

All statistically significant *p*-values (*p* < 0.05) are in bold; Abbreviations: CRAE, central retinal arteriolar equivalent; CRVE, central retinal venular equivalent; AVR, arterio-venous ratio; Stand., standardized; BMI, body mass index; HTN, hypertension; DM, diabetes mellitus.

## Data Availability

The data that were analyzed in this study are available from the corresponding author upon reasonable request.

## Supplementary Materials

The following supporting information can be downloaded at https://www.mdpi.com/article/10.3390/reports9020147/s1. Table S1: Regression analysis with CRAE as dependent variable (fully adjusted model); Table S2: Regression analysis with CRVE as dependent variable (fully adjusted model); Table S3: Regression analysis with AVR as dependent variable (fully adjusted model). References [27,28] are cited in the Supplementary Materials.

## Abbreviations

The following abbreviations are used in this manuscript:

AVR	Arteriolar-to-Venular Diameter Ratio
CRAE	Central Retinal Arteriolar Equivalent
CRVE	Central Retinal Venular Equivalent
