# Supplementary material for: Quantitative Comparison of a Handheld and a Table-Top Fundus Camera for Retinal Microvascular Assessment
Source: Reports (MDPI). 2026 May 11;9(2):147. doi: 10.3390/reports9020147 (PMC13214827; doi:10.3390/reports9020147)
Supplement: Supplementary file 1 [file reports-09-00147-s001.zip › reports-4177677-supplementary.pdf]

Quantitative Comparison of a Handheld and a Table-Top Fundus Camera for Retinal Microvascular Assessment

Lazaros K. Yofoglu[1], Georgios Zervas[2], Christina Konstantaki[2], Chrysoula Moustou[2],

Evaggelia K. Aissopou[1], Petros P. Sfikakis[3], Irini Chatziralli[4],

Kimon Stamatelopoulos[2], Athanase D. Protogerou[1,5*], Antonios A. Argyris[1*]

[1]Cardiovascular Prevention and Research Unit, Clinic/Laboratory of Pathophysiology, School of Medicine, National and Kapodistrian University of Athens, Athens, Greece.
[2]Angiology and Endothelial Pathophysiology Unit, Department of Clinical Therapeutics, Alexandra Hospital, School of Medicine, National and Kapodistrian University of Athens, Athens, Greece.
[3]1st Department of Propaedeutic and Internal Medicine, Laiko Hospital, School of Medicine, National and Kapodistrian University of Athens, Athens, Greece.
[4]2nd Department of Ophthalmology, Attikon Hospital, School of Medicine, National and Kapodistrian University of Athens, Athens, Greece.
[5] Hellenic Foundation for Cardiovascular Health and Nutrition

*Professor Athanase D. Protogerou and Dr. Antonios A. Argyris contributed equally as co-last authors.

Corresponding author:
Professor Athanase D. Protogerou
Cardiovascular Prevention & Research Unit,
Clinic & Laboratory of Pathophysiology
School of Medicine
National and Kapodistrian University of Athens
75, Mikras Asias Street, (Building 16, 3rd floor, room 8)
115 27 Athens, GR
Tel/fax: 0030 210 746 2566, email: aprotog@med.uoa.gr

A. Retinal vessel biomarkers: definitions applied

(a) Central Retinal Arteriolar Equivalent (CRAE) is an expression of the overall arteriolar retinal caliber, summarizing all arteriole diameter measurements into a single biomarker. It is calculated by combining diameter measurements of all arterioles (branches) in a concentric zone around the disc, using a formula, in order to obtain estimates of their trunks and then with the same procedure, combining pairs of trunks until all arterioles have been summarized into a single central retinal arteriolar equivalent (CRAE) [1,2].

(b) Central Retinal Venular Equivalent (CRVE) is considered to be an expression of the overall venular caliber of the retina. Hubbard et al. converted the Parr formula mentioned above and they derived the biomarker central retinal venular equivalent (CRVE) [3].

(c) Arteriolar-to-Venular Diameter Ratio (AVR) is the ratio of CRAE to CRVE [3].

Corresponding References:

[1] Parr JC, Spears GFS. General caliber of the retinal arteries expressed as the equivalent width of the central retinal artery. Am J Ophthalmol 1974;77:472–7. https://doi.org/10.1016/0002-9394(74)90457-7.

[2] Parr JC, Spears GFS. Mathematic relationships between the width of a retinal artery and the widths of its branches. Am J Ophthalmol 1974;77:478–83. https://doi.org/10.1016/0002-9394(74)90458-9.

[3] Hubbard LD, Brothers RJ, King WN, Clegg LX, Klein R, Cooper LS, et al. Methods for evaluation of retinal microvascular abnormalities associated with hypertension/sclerosis in the Atherosclerosis Risk in Communities Study. Ophthalmology 1999;106:2269–80. https://doi.org/10.1016/S0161-6420(99)90525-0.

B. Supplemental Tables

Table S1. Regression analysis with CRAE as dependent variable (fully adjusted model).

	Stand. β	Unstand. β	95% CI	p-value
**Age**	-0.122	-0.166	-0.476 to 0.144	0.291
**Gender**	0.124	5.950	-3.122 to 15.021	0.196
**BMI**	-0.260	-1.299	-2.399 to -0.200	**0.021**
**Smoking**	-0.001	-0.048	-9.773 to 9.678	0.992
**Caffeine**	0.006	0.357	-10.425 to 11.139	0.948
**Alcohol**	-0.235	-11.279	-19.447 to -3.111	**0.007**
**Exercise**	-0.021	-1.140	-9.896 to 7.615	0.796
**Hypertension**	-0.311	-17.927	-31.706 to -4.147	**0.011**
**Diabetes Mellitus**	0.264	25.181	3.101 to 47.262	**0.026**
**Left or Right Eye**	0.045	2.187	-4.846 to 9.219	0.538
**Aurora - Topcon**	-0.523	-25.148	-32.181 to -18.116	**<0.001**

CRAE, central retinal arteriolar equivalent; Stand., standardized; Unstand., unstandardized; CI, confidence intervals; BMI, body mass index.

Table S2. Regression analysis with CRVE as dependent variable (fully adjusted model)

	Stand. β	Unstand. β	95% CI	p-value
**Age**	-0.121	-0.135	-0.385 to 0.116	0.289
**Gender**	0.194	7.617	0.275 to 14.959	**0.042**
**BMI**	-0.118	-0.483	-1.373 to 0.406	0.283
**Smoking**	-0.076	3.380	-4.492 to 11.251	0.396
**Caffeine**	-0.039	-1.953	-10.679 to 6.774	0.658
**Alcohol**	0.106	4.170	-2.441 to 10.780	0.213
**Exercise**	0.181	8.076	0.990 to 15.162	**0.026**
**Hypertension**	-0.259	-12.206	-23.358 to -1.053	**0.032**
**Diabetes Mellitus**	0.363	28.372	10.501 to 46.243	**0.002**
**Left or Right Eye**	-0.006	-0.220	-5.912 to 5.471	0.939
**Aurora - Topcon**	-0.575	-22.620	-28.312 to -16.929	**<0.001**

CRVE, central retinal venular equivalent; Stand., standardized; Unstand., unstandardized; CI, confidence intervals; BMI, body mass index.

Table S3. Regression analysis with AVR as dependent variable (fully adjusted model)

	Stand. β	Unstand. β	95% CI	p-value
**Age**	-0.040	0.000	-0.001 to 0.001	0.755
**Gender**	-0.026	-0.004	-0.036 to 0.028	0.808
**BMI**	-0.269	-0.004	-0.008 to 0.000	**0.033**
**Smoking**	-0.083	-0.014	-0.049 to 0.020	0.413
**Caffeine**	0.041	0.008	-0.031 to 0.046	0.688
**Alcohol**	-0.488	-0.075	-0.104 to -0.046	**<0.001**
**Exercise**	-0.245	-0.043	-0.074 to -0.011	**0.008**
**Hypertension**	-0.241	-0.044	-0.094 to 0.005	0.078
**Diabetes Mellitus**	0.035	0.011	-0.068 to 0.900	0.790
**Left or Right Eye**	0.059	0.009	-0.016 to 0.340	0.475
**Aurora - Topcon**	-0.177	-0.027	-0.520 to -0.002	**0.036**

AVR, arterio-venous ratio; Stand., standardized; Unstand., unstandardized; CI, confidence intervals; BMI, body mass index.
